# On-Demand CMOS-Compatible Fabrication of Ultrathin Self-Aligned SiC Nanowire Arrays

**DOI:** 10.3390/nano8110906

**Published:** 2018-11-05

**Authors:** Natasha Tabassum, Mounika Kotha, Vidya Kaushik, Brian Ford, Sonal Dey, Edward Crawford, Vasileios Nikas, Spyros Gallis

**Affiliations:** 1 Colleges of Nanoscale Sciences and Engineering, SUNY Polytechnic Institute (SUNY Poly), Albany, NY 12203, USA; ntabassum@sunypoly.edu (N.T.); mkotha@sunypoly.edu (M.K.); vkaushik@sunypoly.edu (V.K.); Brford@sunypoly.edu (B.F.); sonal_dey@avs.org (S.D.); vnikas@sunypoly.edu (V.N.); 2 GLOBALFOUNDRIES Corp., East Fishkill, NY 12533, USA; Edward.Crawford@globalfoundries.com

**Keywords:** silicon carbide, ultrathin nanowires, nanofabrication, self-aligned nanowires, telecom wavelengths, quantum photonics

## Abstract

The field of semiconductor nanowires (NWs) has become one of the most active and mature research areas. However, progress in this field has been limited, due to the difficulty in controlling the density, orientation, and placement of the individual NWs, parameters important for mass producing nanodevices. The work presented herein describes a novel nanosynthesis strategy for ultrathin self-aligned silicon carbide (SiC) NW arrays (≤ 20 nm width, 130 nm height and 200–600 nm variable periodicity), with high quality (~2 Å surface roughness, ~2.4 eV optical bandgap) and reproducibility at predetermined locations, using fabrication protocols compatible with silicon microelectronics. Fourier transform infrared spectroscopy, X-ray photoelectron spectroscopy, ultraviolet-visible spectroscopic ellipsometry, atomic force microscopy, X-ray diffractometry, and transmission electron microscopy studies show nanosynthesis of high-quality polycrystalline cubic 3C-SiC materials (average 5 nm grain size) with tailored properties. An extension of the nanofabrication process is presented for integrating technologically important erbium ions as emission centers at telecom C-band wavelengths. This integration allows for deterministic positioning of the ions and engineering of the ions’ spontaneous emission properties through the resulting NW-based photonic structures, both of which are critical to practical device fabrication for quantum information applications. This holistic approach can enable the development of new scalable SiC nanostructured materials for use in a plethora of emerging applications, such as NW-based sensing, single-photon sources, quantum LEDs, and quantum photonics.

## 1. Introduction

As the field of semiconductor nanowires (NWs) has become one of the most active and relatively mature research areas, heightened interest in the synthesis, characterization, and applications of these NWs has become prevalent within the scientific community. The unique properties of ultrathin NWs, resulting from their reduced dimensionality coupled with their tunable properties and surface functionalization, make them promising for various applications in the field of electronics [1], optics [2], biological sciences [3,4,5], medical diagnosis [6], energy harvesting [7], and ultra-high nanosensing [8]. Furthermore, great efforts have been focused on the development of nanostructured materials that may be employed in emerging quantum applications, such as quantum imaging and sensing, and quantum photonics [9,10].

Top-down and bottom-up approaches are the two basic paradigms of nanofabrication. The top-down approach refers to the etching of bulk material to sculpt nanostructures. This has the primary advantage of direct assembly after processing, as the density and spatial location of the resulting nanostructure are defined by the process. However, top-down fabrication is heavily dependent on lithographical patterning of the desired features and thus, fabricating features below 20 nm becomes difficult and costly, and the composition of the resulting nanostructured materials is limited to the bulk materials from which they are fabricated.

The bottom-up approach allows for the fabrication of a wide range of materials by assembling the required subcomponents in an additive fashion. Common techniques in this paradigm include vapor-liquid-solid [11], template-assisted electrochemical deposition [12], and solution-based growth strategies [13]. The advantages of bottom-up techniques include the synthesis of a wide range of both inorganic and organic materials, synthesis of heterostructures, such as axial and radial core/shell structures, and the ability to dope in situ. The main limiting challenge commonly faced by this paradigm is the required deterministic assembly, which involves control over the density, orientation, spacing, and placement of the individual NWs, and their integration into large-scale arrays with high scalability and reproducibility [14].

Deterministic assembly of NW arrays is essential for the mass production of electronic nanodevices and the creation of practical nanoscale-based systems for the fabrication of functional interconnected nanosystems [14]. Furthermore, the realization of devices in the emerging field of quantum technologies requires innovative nanomaterial architectures, where the optical and quantum properties of emission centers can be deterministically engineered [10].

Silicon Carbide (SiC) is a silicon-based wide band-gap material, which exhibits strong mechanical properties and is chemically inert. These properties have led to the employment of SiC in a variety of applications due their stability within a multitude of environments: In high-temperature energy conversion devices and in chemically corrosive and high shock environments [15,16]. SiC-based field-effect transistors (FETs) have demonstrated operation for thousands of hours under high-temperature conditions [17] and SiC NWs have been used in hydrogen sensors [18]. Furthermore, owing to its high biocompatibility [19], SiC is used in the biomedical field for coating implants, as SiC nanostructures enhance cell proliferation and accelerate tissue reconstruction [20,21]. In the field of nanobiotechnology, SiC NW-FETs have demonstrated the ability to detect DNA hybridization [22]. Moreover, naturally occurring Si and C in SiC has almost negligible magnetic moment [23], which is a necessary requirement for hosting quantum emitters with reduced optical decoherence caused by nuclear and electronic spin fluctuation [24]. In that regard, SiC nanophotonic structures have been recognized as promising systems for several applications in quantum technologies [10].

Herein, the current investigators present an innovative and straightforward synthesis route for SiC NW arrays. This synthesis route allows for ultrathin self-aligned NWs to be fabricated without the use of a lithographic-pattern-transfer technique. This fabrication scheme overcomes obstacles faced by top-down and bottom-up approaches, which typically result in high surface defect density states, due to the dry-etch step or random orientation and size, non-specific positioning, or requires transfer to another substrate and subsequent fabrication steps. Ultrathin array NWs (≤20 nm) are advantageous for NW array-based biosensors with high sensitivity, as the reduced size allows for full gating of the NW by the charged species of interest [8]. Ultrathin SiC NW allows for higher mechanical strength [25] and reduced bulk-defect density [26], which is particularly beneficial for the emission of color centers [27,28]. This synthesis strategy may serve as a common experimental platform to investigate multiple SiC NW-based emerging technologies, such as NW-based sensing, single photon sources, quantum LEDs and quantum photonics. To this end, we have extended the above mentioned fabrication process to host erbium ions in SiC NW arrays. The integration scheme allows us to control the locations of erbium ions in SiC NWs and to modify the spontaneous emission properties of these ions. Both are important components towards potential device applications in the emerging field of quantum photonics.

## 2. Materials and Methods

**Figure 1:**
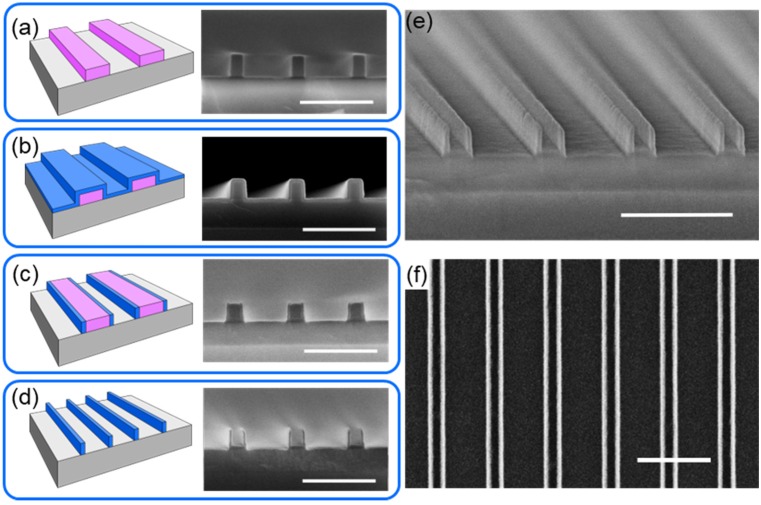
**Nanofabrication of ultrathin self-aligned nanowires (NW) array.** (**a**) Si wafer (gray) was spin-coated with hydrogen silsequioxane (HSQ) followed by exposure and development yielding a ribbon array (pink) with width and pitch ranging from 50 nm to 150 nm and from 200 nm to 600 nm, respectively; (**b**) ultrathin conformal silicon carbide (SiC) layer (blue) was deposited using thermal CVD; (**c**) ultrathin conformal SiC layer was etched open to expose the HSQ ribbon array using inductively coupled plasma reactive ion etching (ICP-RIE); (**d**) removal of the HSQ ribbon array was done by wet etch in buffered hydrofluoric acid (BHF), yielding SiC NW arrays with 20 nm critical dimension (width) NWs. Cross-section SEM images are shown after corresponding steps; (**e**) tilted cross-section and (**f**) top-down SEM image of the SiC NW array. Scale bar in all SEM images is 500 nm.

A simplified schematic representation with corresponding scanning electron microscope images (SEMs) of the growth strategy for ultrathin self-aligned SiC NW arrays is shown in Figure 1. First, ribbon arrays (250 × 250 µm^2^) of hydrogen silsequioxane (HSQ) negative-tone resist were fabricated on clean Si (100) substrates using electron-beam lithography (EBL). The Si substrate was spin-coated with HSQ (6 wt.% HSQ in methyl isobutyl ketone (MIBK)), followed by a soft-bake at 80 °C for 4 min prior to exposure. The HSQ resist layer was exposed with 100 kV beam using VB300 EBL system (Vistec Electron Beam GmbH, Jena, Germany) or with a 50 kV beam using Voyager system (Raith GmbH, Dortmund, Germany). Exposed resist layer then developed in 2.38 wt.% of tetra methyl ammonium hydroxide (TMAH), yielding an HSQ ribbon array (Figure 1a). Electron-beam lithography was used for rapid prototyping while minimizing expenses; however, with the proper photomasks and processes, the fabrication scheme presented is seamlessly compatible with standard photolithography by using a thin oxide or nitride film instead of the resist layer (Figure 1a).

Nanowire synthesis was conducted using a well-controlled thermal chemical vapor deposition (CVD) process, which has been reported previously for the synthesis of silicon carbide (SiC) and silicon oxycarbide (SiC:O) [29,30,31]. For this work, ultrathin (10 to 40 nm) SiC or SiC:O was deposited onto the HSQ ribbon array, followed by a thermal anneal for 1 hour in forming gas (5% H_2_, 95% Ar) at a temperature ranging from 900 to 1200 °C. The thickness of the SiC conformal layer synthesized onto the HSQ ribbon array (Figure 1b) defines the critical dimension (width) of the NWs, hence the NW width is solely dependent on the deposition process and not any lithographic transfer/post-material-synthesis etching.

The Si and C single-source oligomer used was CVD-742 (1,1,3,3-tetramethyl-1,3-disilacyclobutane, Starfire Systems) along with forming gas (5% H_2_, 95% N_2_) as a dilution gas. The SiC conformal layer was synthesized at 800 °C while system pressure was maintained at 1.0 Torr with a precursor flow rate fixed at 10 sccm. For optional oxygen doping (synthesis of SiC:O), synthesis parameters (temperature, pressure, precursor flow rate) were identical to the SiC synthesis with the exception of dilution gas and the use of a co-reactant, which were ultra-high purity Ar and O_2_ respectively.

After synthesis of the conformal layer of SiC or SiC:O on the HSQ ribbon array, inductively coupled plasma reactive ion etching (ICP-RIE) was performed to remove the undesired material (ultrathin film between and on top of HSQ ribbons) and to expose the HSQ ribbon array for subsequent wet-etch removal (Figure 1c). ICP-RIE was performed at 7 mTorr using a ratio of 90:10 CHF_3_:O_2_ for 10–15 seconds. After etching, the NW array fabrication was completed upon removal of the HSQ ribbon array by dipping the sample in buffered hydrofluoric acid (BHF) for 5 min (Figure 1d). Representative cross-section and top-down SEM images of the resulting nanowire array structure are shown in Figure 1e,f.

For photoluminescence (PL) measurements, we used a home-built micro-PL (µPL) system–composed of an argon laser (model: Beamlock 2065, Spectra-Physics, Santa Clara, CA, USA), a dichroic mirror (DM), a 50× objective lens, a scanning nano-stage (1 nm resolution) for sample positioning, a fiber coupled FLSP920 spectrometer (Edinburgh Instruments, Livingston, UK) and an InGaAs detector.

## 3. Results and Discussion

### 3.1. Nanofabrication

**Figure 2:**
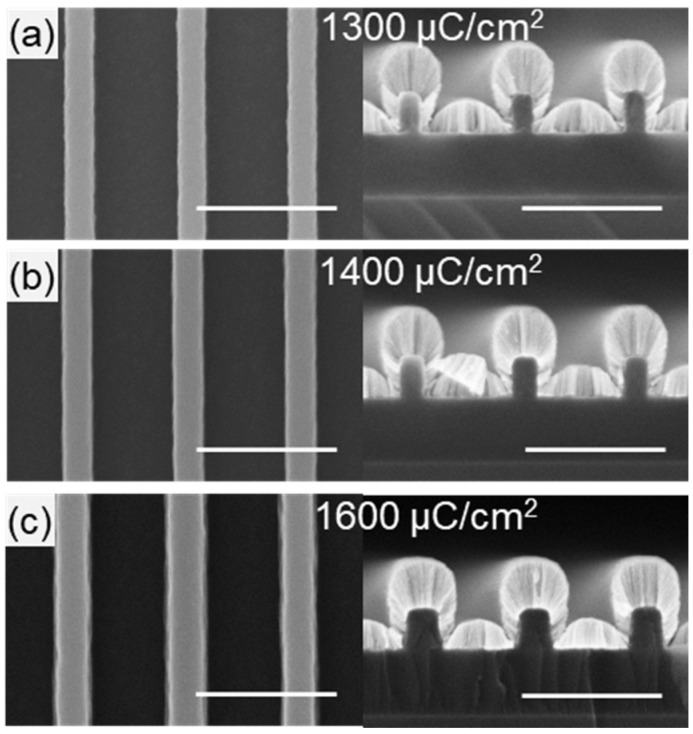
**Dose array for HSQ ribbon array.** Top-down (left) and cross-section (right, with metallization for imaging contrast) SEM images of approximately 100 nm wide HSQ ribbons after exposure at (**a**) 1300 μC/cm^2^; (**b**) 1400 μC/cm^2^ and (**c**) 1600 μC/cm^2^. For all the SEM images shown, scale bar is 500 nm.

**Figure 3:**
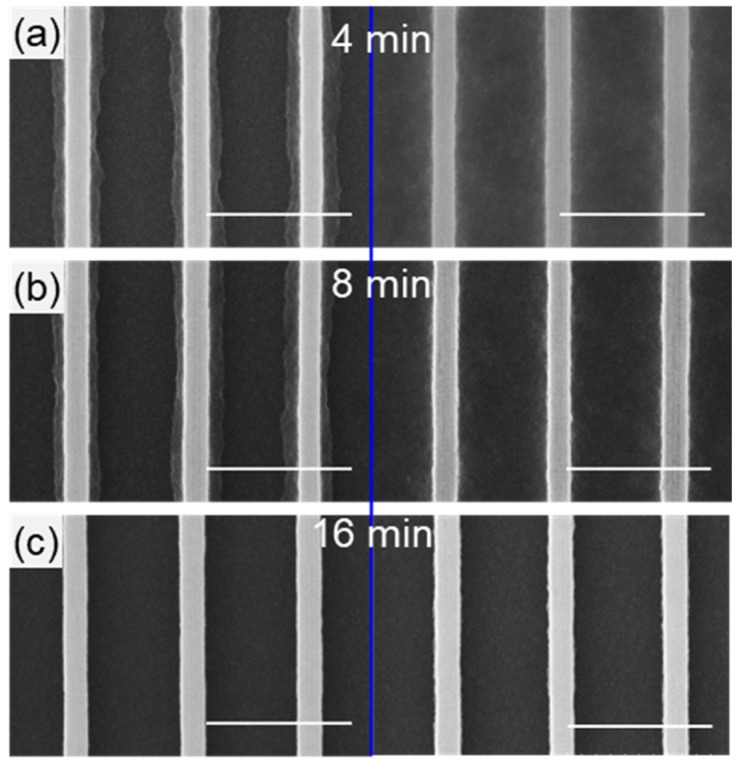
**Development time for HSQ ribbon array.** Comparison of development time at the edge (left column) and center (right column) of the HSQ ribbon array after (**a**) 4 min; (**b**) 8 min, and (**c**) 16 min of development time. Scale bar is 500 nm for all the SEM images shown.

Lithography parameters for the HSQ ribbon array were identified by performing a dose array study from 1200 to 1750 μC/cm^2^ on a representative layout for the ribbon array with 100 nm wide lines with a pitch of 400 nm. A dose of 1400 µC/cm^2^ was observed to best replicate the designed dimensions (Figure 2b). A lower dose of 1300 µC/cm^2^ (Figure 2a) resulted in thinner ribbons indicating under-exposure and a higher dose at 1600 µC/cm^2^ (Figure 2c) resulted in thicker ribbons with flared-out base indicating over-exposure. The optimal development time for the HSQ ribbon array was found by increasing the development time in 4-min increments. Shown in Figure 3c, 16 min was required for complete removal of the HSQ residue between ribbons. It is worth noting that the HSQ residue was removed more quickly at the edge of the ribbon array (Figure 3, left column) compared to the center (Figure 3, right column).

By using the proposed nanofabrication approach, where the critical dimension of the NWs is defined by a well-controlled deposition process, production complexity can be reduced. Furthermore, the NW height can be controlled by changing the HSQ thickness with different parameters during spin-coating. Most importantly, this integration scheme can be material-invariant with proper etch and deposition techniques.

**Figure 4:**
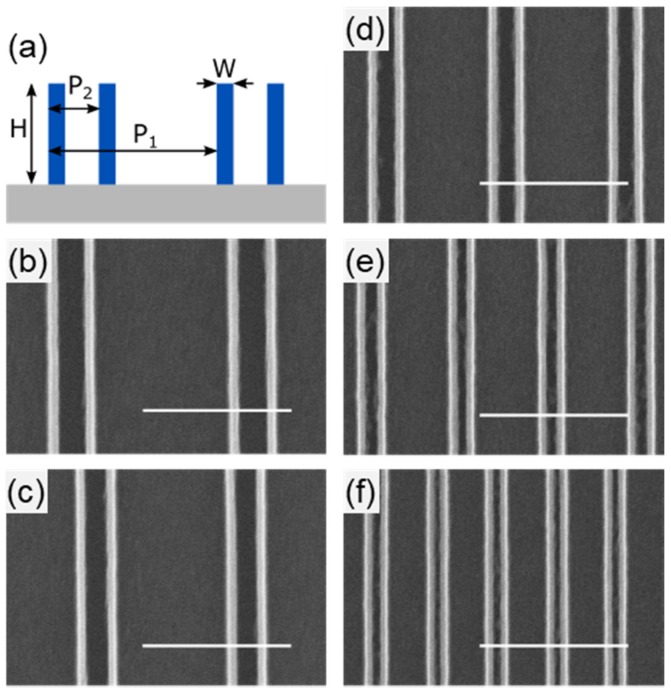
**Modulation of NW array pitch (P_1_) and sub-pitch (P_2_).** (**a**) Schematic representation of the resulting NW array structure with pitch (P_1_) and sub-pitch (P_2_), and NW height (H) and width (W) by modifying the layout of the HSQ ribbon array. Arrays of 20 nm (width, W) SiC NWs with a P_1_ to P_2_ ratio of 4:1 were fabricated. After nanofabrication, SEM images were collected for P_1_:P_2_; (**b**) 600:150; (**c**) 500:125; (**d**) 400:100; (**e**) 300:75; and (**f**) 200:50 (all numbers are in nm). Scale bars in all the SEM images are 500 nm.

Flexibility of the growth strategy was explored by creating HSQ ribbon arrays with a pitch-to-ribbon-width ratio of 4:1. The pitch is denoted as P_1_ and the ribbon width, which becomes sub pitch of the resulting NWs is denoted as P_2_. Ribbon arrays were fabricated with dimensions of P_1_:P_2_-600:150, 500:125, 400:100, 300:75, and 200:50 (all numbers are in nm). A schematic depiction of the resulting structures is shown in Figure 4a with corresponding SEMs in Figure 4b–f. The critical dimension (width) and the spacing of the NWs can be modulated by adjusting the deposition time and customizing the lithography accordingly based on specific application requirements, such as increased spacing for subsequent processing.

### 3.2. Structural, Compositional, Optical, and Morphological Properties

Quality of the synthesized SiC nanomaterial, i.e., stoichiometry, crystal phase, surface roughness, defect density etc., are very important to investigate for any practical device application. Different characterization analyses, such as Fourier transform infrared spectroscopy (FTIR), X-ray photoelectron spectroscopy (XPS), ultraviolet visible spectroscopic ellipsometry (UV-VIS-SE), atomic force microscopy (AFM), high-resolution scanning transmission electron microscopy (HR-STEM) and X-ray diffractometry (XRD) were systematically carried out to assess and optimize the deposition and post-deposition process parameters towards achieving high-quality SiC nanowires.

**Figure 5:**
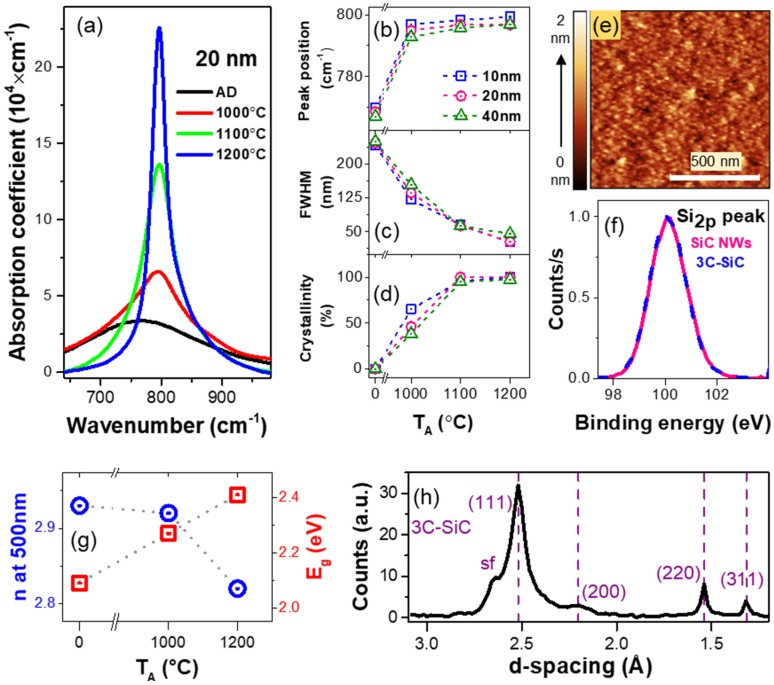
**Structural, compositional and optical analysis of the synthesized SiC.** (**a**) The Si–C stretching mode of the as-deposited (AD) and annealed 20-nm SiC at 1000, 1100, and 1200 °C in FG (5% H2, 95% Ar); (**b**) peak position of the Si–C stretching mode; (**c**) full width at half maximum (FWHM) of the Si–C stretching mode; and (**d**) crystalline fraction in materials as a function of annealing temperature, T_A_. Error bars are not depicted as the errors are smaller than the symbol size; (**e**) representative atomic force microscopy (AFM) image of the surface of 20 nm SiC ultrathin film after 1100 °C anneal; (**f**) X-ray photoelectron spectroscopy (XPS) data of Si 2p peak from synthesized SiC and 3C-SiC control sample; (**g**) refractive index, n at 500 nm and Tauc optical gap, E_g_ vs. T_A_ for SiC, where errors are smaller than the symbol size; (**h**) X-ray diffractometry (XRD) pattern of 1200 °C-annealed SiC. The dashed lines correspond to the d-spacing values of (111), (200), (220) and (311) planes of 3C-SiC phase; sf, stacking faults.

FTIR spectroscopy showed a single strong absorption peak at ~760 cm^−1^ for all synthesized SiC, corresponding to Si–C stretching mode [32,33]. As shown in Figure 5a, upon increasing annealing temperature, *T*_A_, three notable changes in the absorption spectra were observed: (1) A shift in peak position towards 800 cm^−1^ (Figure 5a), which corresponds to the stretching mode of Si–C bond in crystalline SiC [33,34], (2) substantial narrowing of the full width at half maximum (FWHM) (Figure 5b) and, (3) a change in line shape from Gaussian to Lorentzian. The line shape of the Si–C stretching mode of the as-deposited (AD) SiC was fitted with a Gaussian function of ~265 cm^−1^ FWHM, indicating a Gaussian distribution of bond lengths and angles, which characterizes the amorphous phase. Moreover, a transformation of the line shape to Lorentzian, corresponding to a more uniform environment of Si–C bonds, suggest the formation of crystalline SiC in the materials [35,36]. Upon annealing, the FWHM decreased to ~30 cm^−1^, comparable to values for high-quality SiC [37].

Furthermore, to isolate the contribution of amorphous and crystalline phase, we deconvoluted the FTIR absorption spectra of the AD and annealed SiC materials into Gaussian (G) and Lorentzian (L) components, then the areas of each component were used to determine the crystalline fraction by calculating L/(L + G) (Figure 5d). For example, for the 10 and 20 nm ultrathin films, it was observed that annealing at 1100 °C for one hour resulted in complete crystallization as the absorption band could be fit using a single Lorentzian. Increasing the annealing temperature further only decreased the FWHM. The overall transformation of FTIR absorption spectra of Si–C mode with T_A_ suggests a high-quality and degree of crystalline environment of the synthesized SiC.

AFM was performed on SiC ultrathin films in order to assess the surface morphology. A representative AFM micrograph of a representative 1100 °C-annealed SiC sample is shown in Figure 5e, where the mean surface roughness was determined to be ~2 Å, which is comparable to a polished Si wafer [38].

We performed compositional analysis of the annealed SiC via XPS. The ratio of silicon and carbon was found to be 50:50 within <1% experimental uncertainty. Additionally, strong overlap for the Si 2p binding energy between the annealed SiC and a 3C–SiC standard at ~100.3 eV confirms the chemical bonding of the synthesized materials and NWs are indeed Si–C (Figure 5f). The absence of XPS Si 2p peak around ~98.5 and ~103 eV, corresponding to Si–Si and Si–O bonds respectively [32], also ruled out the possibility of silicon or carbon nanocluster formation, as well as any oxidation.

The refractive index *n*, and Tauc optical gap *E*_g_, of the SiC nanomaterials further elucidate the quality of synthesized materials. We extracted both parameters from UV-VIS-SE measurements (SOPRALAB, Semilab Co. Ltd., Budapest, Hungary). A decrease in refractive index upon annealing at higher temperatures was observed (Figure 5g), approaching the reference value for 3C–SiC (~2.7) [39]. *E*_g_ values were calculated using Tauc’s law *αE* = *B*(*E* − *E_g_*)^2^, where *α* is the absorption coefficient, *B* is the slope (which is inversely proportional to the band tail width), *E* is the photon energy, and *E_g_* is the optical bandgap [29]. *E_g_* increased with higher annealing temperatures, approaching the reference value of ~2.4 eV for 3C–SiC [40].

To determine the crystalline phase of the material, XRD analysis was done on different samples. The grazing incidence XRD (GIXRD) experiments were done at the Cornell High Energy Synchrotron Source (CHESS) using X-rays of energy 11.4 keV. The details of the experimental setup can be found elsewhere [41]. The vertical lines in the grazing incidence d-spacing map (GIDSM) confirmed the polycrystalline nature of the material. The GIDSM data were integrated to obtain an intensity versus d-spacing GIXRD plot for a 40 nm 1200 °C-annealed SiC (Figure 5h). The indexing was done with JCPDS #00-029-1129 (SiC, FCC, space group #216; a = 4.35890 Å). The peak positions align well with 3C–SiC phase. The d-spacing value at 2.52, 2.2, 1.54 and 1.32 (corelates to 2*θ* = 35.6°, 40.9°, 60.0°, and 70°) correspond to the (111), (200), (220), and (311) planes observed in 3C–SiC [42]. Predominant peak of (111) plane signifies that the 3C-SiC nanocrystals have a preferred orientation of growth. We used both Scherrer formula [34] and Williamson-Hall plot [43] to estimate the average grain sizes of the annealed SiC. From Scherrer formula, we found the grain size to be around 3–5 nm. The calculated grain size from Williamson-Hall plot’s intercept was found to be approximately 4.5 nm. Peak broadening may be attributed to strain or size-confinement, and the shoulder at 2.64 Å may be due to stacking faults in SiC [33,43].

**Figure 6:**
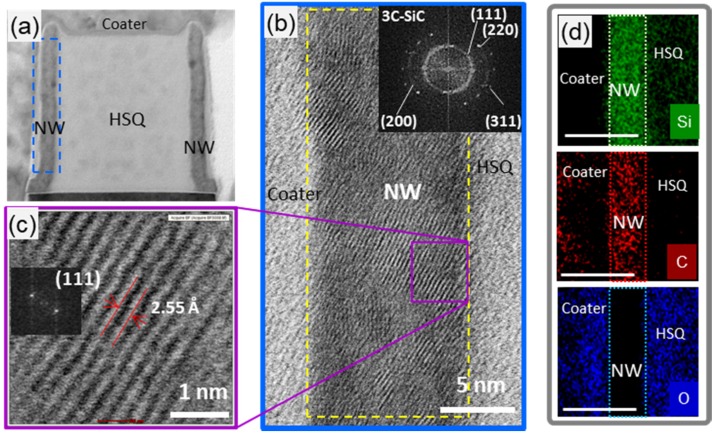
**Transmission electron microscopy (TEM) analysis of SiC NW array with P_1_:P_2_ = 400:100 nm** (**a**) Representative TEM image of a pair of 10 nm (width, W) SiC NWs; (**b**) High-resolution TEM image of a single nanowire. Inset: A fast Fourier transform (FFT) of the yellow framed area in (**b**), showing spotted rings with approximate radii of 2.5, 2.22, 1.55 and 1.34 Å corresponding to (111), (200), (220), (311) planes of 3C–SiC; (**c**) HRTEM (high-resolution scanning TEM) image from the purple framed area in (**b**) showing the (111) orientation, which is confirmed by the FFT in inset; (**d**) Elemental analyses of an area surrounding a single 10 nm NW.

HR-STEM along with energy-dispersive spectroscopy (EDS) studies were also performed, as shown in Figure 6. The images showed that the NW is polycrystalline with small grains size of average ~5 nm (Figure 6b), confirming the calculated grain size from XRD. Indexing of the digital diffraction pattern was obtained by FFT (Fast Fourier Transform). FFT showed ring patterns with radii of 2.51 Å, 2.22 Å, 1.55 Å and 1.34 Å corresponding well to the inter-planar spacings of cubic 3C-SiC (Figure 6b). The TEM image and the discontinuity of the observed ring at certain angles suggest a preferential growth direction of the 3C-SiC grains in ultrathin NWs [44]. The spacing between the lattice fringes of a single grain was, on average, ~2.55 Å (Figure 6c), which is close to d-spacing, ~2.52 Å, of the (111) plane of 3C–SiC [45]. FFT on that single grain showed only two bright spots with ~2 × 2.51 Å distance, suggesting a single crystalline 3C–SiC. The EDS maps confirm the presence of Si and C in the NW with no oxidation.

### 3.3. Deterministic Ion Integration Into NW Arrays

**Figure 7:**
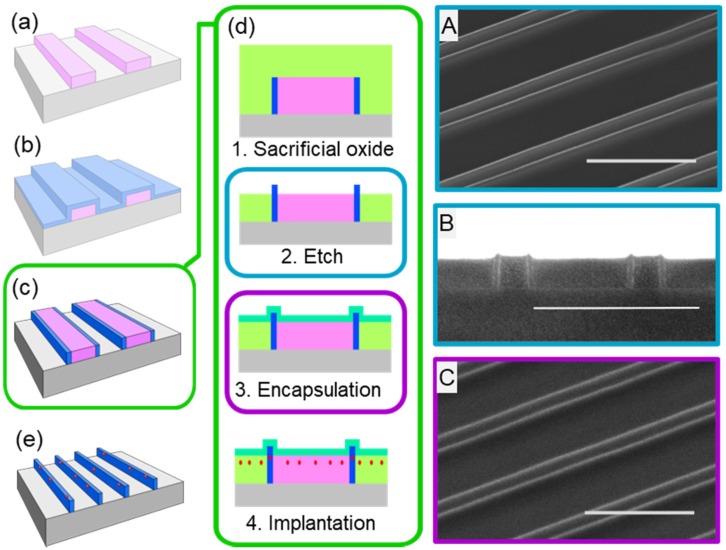
**Integration scheme for controlled ion implantation into NW array.** Schematics (**a**)–(**c**) correspond to Figure 1a–c; (**d**) schematic representation of the process steps involved in the ion implantation; green box continued from (**c**): (1) Thick sacrificial silicon oxide layer (light green) deposition. (2) Sacrificial oxide planarized and recessed below the tips of the NWs. Corresponding top-down SEM shown in **A** with cross-section in **B** (scale bar is 500 nm). (3) Ultrathin encapsulation silicon oxide layer (turquoise) deposition. Corresponding top-down SEM shown in **C** (scale bar is 500 nm). (4) Erbium ions (red) implantation into the structure; (**e**) erbium-doped NW array after removal of the sacrificial oxide and HSQ ribbon array.

We extended the growth strategy for synthesizing self-aligned SiC NW arrays and implemented it for the deterministic placement of erbium (Er) ions into the NW arrays (Figure 7). The proposed integration scheme is not specific to erbium ions, opening up a great potential for the use of SiC nanophotonic structures for applications in quantum information and quantum photonics [10,46]. The extended fabrication scheme begins after the ICP-RIE to expose the HSQ ribbon array and before the wet-etch step previously discussed (see Figure 7c). First, a ~200 nm thick sacrificial oxide was deposited using thermal CVD. The oxide was planarized using chemical mechanical planarization and was then recessed to expose the top of the NWs using ICP-RIE followed by a wet-etch in BHF for 10 seconds, as shown in Figure 7(d-1,2). Following the oxide recession, an encapsulation oxide was deposited targeting 15 nm thickness prior to ion implantation (Figure 7(d-3,4)). The encapsulation oxide (Figure 7(d-3,4)) serves two purposes: (1) It allows for tailoring the target implantation depth of the ions into the NWs, and (2) it protects the NW surface from ion implantation damage. Erbium ion implantation was performed using an Extrion 400 Ion Implanter, targeting 45 nm implantation depth. The doses of erbium ions were varied between 1 × 10^13^–1 × 10^14^ cm^2^, a typical range for characterizing emission behavior of ions minimizing inter-ionic interaction [47]. After the ion implantation, the samples were wet-etched in BHF for five minutes to remove the encapsulation oxide, sacrificial oxide, and HSQ ribbon array, and annealed at 900 °C for one hour in ultra-high purity Ar to optically activate the Er ions (Er^3+^) based on our group’s previous study [48].

### 3.4. Photoluminescence Properties

**Figure 8:**
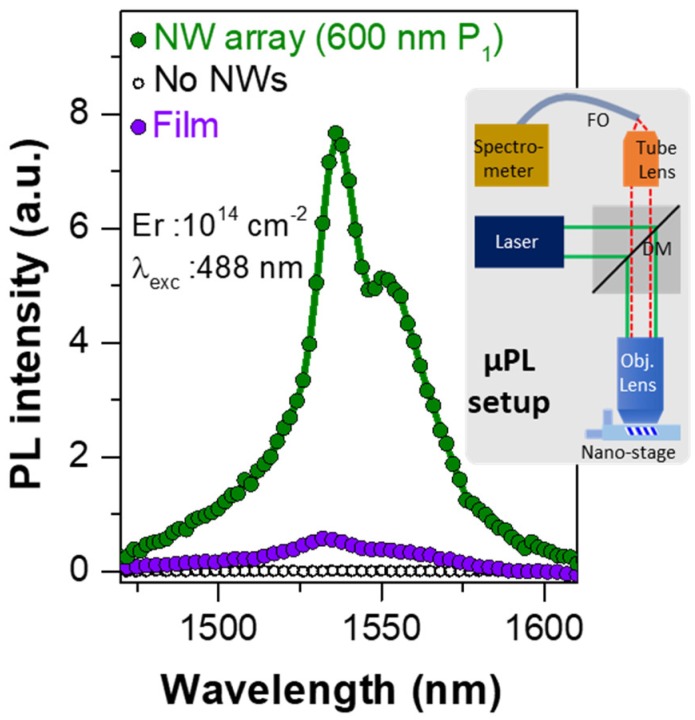
**Room temperature Er-induced PL spectra.** Er^3+^ PL ~1540 nm, corresponding to the intra-4*f* transition (^4^*I*_13/2_ → ^4^*I*_15/2_) of Er^3+^ ions, from 20 nm SiC:O NW arrays with P_1_:P_2_-600:150 nm, with no detectable Er^3+^ PL from the region outside the NWs (white circles). For comparison the Er^3+^ PL from a thin-film control is also shown with same Er dose (Er: 10^14^ cm^−2^ dose, 488 nm excitation). The Er^3+^ PL intensity of the thin-film was normalized to the effective area of the NW array [42,43]. Inset: A simplified schematic diagram of the home-built µPL setup composed of an argon laser, objective lens, dichroic mirror (DM), tube lens, fiber optic (FO) coupled to a spectrometer, and an InGaAs detector (see Materials and Methods).

To assess the proposed deterministic ion integration scheme and the potential of NW arrays for controlling the emission properties of Er^3+^ ions, room-temperature steady-state photoluminescence (PL) measurements were carried out on Er-doped SiC:O NW array structures. As shown in Figure 8, we observed a strong Er-induced PL emission around 1540 nm, which is the telecommunication C-band wavelength used in optical fibers, from Er-doped SiC:O NW, with no detectable Er^3+^ PL from the region outside the NWs (white circled points). Er-induced PL spectra ~1540 nm corresponds to the intra-4f transition (^4^I_13/2_ → ^4^I_15/2_) of Er^3+^ ions, which are effectively shielded by the outer 5s and 5p electrons, resulting photostable spectra independent of annealing temperature or ambient [47,48]. Furthermore, an appreciable enhancement of the Er-induced PL was observed in the NW array structure compared to its thin-film counterpart. This enhancement can be attributed to an increase in the emission-extraction efficiency in the SiC photonic crystal structure (created by the periodic arrays of NWs), resulting from the photonic bandgap effect [46,49]. Additionally, in the case of well-passivated ultrathin NWs a reduced bulk defect-density is expected within the ion’s recombination volume contributing to the observed enhanced PL [26,30]. Further details pertaining to the Er^3+^ PL behavior in SiC NW-based photonic structures are reported elsewhere [49].

## 4. Conclusions

In conclusion, herein we report a novel nanofabrication for synthesizing ultrathin self-aligned SiC or SiC:O NW arrays with on-demand positioning and tailored properties. This nanofabrication can enable the synthesis of NW arrays of a wide variety of materials, thus facilitating the study of nanostructured materials, which are difficult to produce by typical methodologies. Most importantly, this synthesis route allows for ultrathin NWs to be fabricated without the use of a lithographic-pattern-transfer technique. Additionally, we describe a fabrication scheme to deterministically integrate erbium ions into ultrathin SiC NW arrays. The high room-temperature Er^3+^ telecom-wavelength PL intensity observed from such NW arrays reveal the integration benefits of our novel nanofabrication scheme. This approach can facilitate the development of new scalable SiC NW-based systems, which can be modified towards NW-based sensing, single-photon emission, and quantum photonics applications.
